# Supplementary material for: Timeliness of routine childhood vaccination in low- and middle-income countries, 1978–2021: Protocol for a scoping review to map methodologic gaps and determinants
Source: PLoS One. 2021 Jun 17;16(6):e0253423. doi: 10.1371/journal.pone.0253423 (PMC8211249; doi:10.1371/journal.pone.0253423)
Supplement: Attachment — Submitted filename: JK Minor Comments- Timeliness Paper.docx [file pone.0253423.s003.docx]

JK Minor Comments:

Recommendations

To increase usefulness of the paper and more comprehensive opportunities for future studies, a 6^th^ review question may be included:

What determinants or factors contributing to untimely childhood vaccination have identified but not studied in LMICs?
