# Supplementary material for: Timeliness of routine childhood vaccination in low- and middle-income countries, 1978–2021: Protocol for a scoping review to map methodologic gaps and determinants
Source: PLoS One. 2021 Jun 17;16(6):e0253423. doi: 10.1371/journal.pone.0253423 (PMC8211249; doi:10.1371/journal.pone.0253423)
Supplement: Attachment — Submitted filename: Response to Reviewers__PONE-D-21-12294.docx [file pone.0253423.s004.docx]

**Journal requirements**

|  | **Journal requirements** | **Authors’ response** |
| --- | --- | --- |
| 1. | Please ensure that your manuscript meets PLOS ONE's style requirements, including those for file naming. The PLOS ONE style templates can be found [HERE](https://journals.plos.org/plosone/s/file?id=wjVg/PLOSOne_formatting_sample_main_body.pdf) and [HERE](https://journals.plos.org/plosone/s/file?id=ba62/PLOSOne_formatting_sample_title_authors_affiliations.pdf) | Thanks for providing the guidelines. We have revised the manuscript to meet the PLOS ONE’s style requirement. All edits are in track changes in the document marked ‘’Revised Manuscript with Track Changes’. |
| 2. | Please review your reference list to ensure that it is complete and correct. If you have cited papers that have been retracted, please include the rationale for doing so in the manuscript text, or remove these references and replace them with relevant current references. Any changes to the reference list should be mentioned in the rebuttal letter that accompanies your revised manuscript. If you need to cite a retracted article, indicate the article’s retracted status in the References list and also include a citation and full reference for the retraction notice | We have reviewed the reference list as requested and can confirm that it is complete and correct. Specifically, we did not make any changes to the reference list and we have not cite any papers which have been retracted. |

**Reviewers' comments**

|  | **Comments for the Authors (Reviewer #1)** | **Authors’ response** |
| --- | --- | --- |
| 1. | Apart from the one comment and suggested addition, the paper is sound, cohesive, relevant and well presented. | We thank the reviewer for the time spent in reviewing our manuscript and for providing very positive comments about our scoping review protocol.  We have provided a point-by-point response to the reviewers’ comment below. |
|  | To increase usefulness of the paper and more comprehensive opportunities for future studies, a 6^th^ review question may be included:  What determinants or factors contributing to untimely childhood vaccination have identified but not studied in LMICs? | We appreciate the reviewer for this thoughtful comment and for recommending the addition of an additional question to the scoping review protocol. While we agree with the reviewer in principle, we did not include this specific question for the reasons we have explained below.  Any determinants or factors contributing to untimely routine childhood vaccinations that have been identified but was not studied in LMICs can only be identified after the completion of this scoping review. We cannot at this point identify such determinants without first conducting a scoping review of the literature on timeliness of vaccinations across LMIC and HIC. Additionally, the included papers and manuscript will not primarily have that information (i.e., each paper will not tell us ‘which determinants already identified that was not studied’). Thus, this question can only be answered deductively after completing the full review. We aim to thoroughly contextualise such factors in the discussion section of the full manuscript by comparing our findings (i.e., the determinants of timeliness identified from our scoping review) to those from studies in HICs. |
